# Genetic Diagnosis in Children with Developmental Delay

**DOI:** 10.3390/children11060669

**Published:** 2024-05-30

**Authors:** Kun-Long Hung

**Affiliations:** 1 Department of Pediatrics, Fu-Jen Catholic University Hospital, New Taipei City 243, Taiwan; klhung@ms10.hinet.net or A00123@mail.fjuh.fju.edu.tw; Tel.: +886-2-8512-8704; Fax: +886-2-2904-6422; 2 School of Medicine, Fu-Jen Catholic University, New Taipei City 242, Taiwan

## 1. Introduction

Developmental delay (DD) has a great impact on children at the developmental stage, and is often manifested by varying degrees of motor delays, intellectual disabilities, and other defects. Diagnosis of DD mainly depend on clinical evaluation and standard testing. Global developmental delay (GDD) and intellectual disability (ID) are two common diagnoses for physicians in caring pediatric patients with neurodevelopmental disorders [1]. The incidence of GDD in children is estimated to be 3 to 5 percent and the incidence of ID is 2 to 3 percent [2]. The etiology of GDD/ID is quite heterogeneous, involving both acquired and inherited causes. Brain hypoxia, metabolic disorders, infections, maternal diseases, and prenatal exposure to various toxins during pregnancy account for a certain percentage of GDD/ID. The genetic causes are found in nearly half of unexplained GDD/IDs [3,4], which may include chromosomal abnormalities, copy number variants (CNVs), as well as monogenic disorders with over 1000 genes being identified [5,6]. The application of genetic testing can help to find the etiologies of unexplained GDD/ID and significantly enhance the diagnostic rate. In order to share the latest research progress, a Special Issue “Genetic Diagnosis of Children with Developmental Delay” was launched. After rigorous review, a total of six papers were finally accepted from 10 manuscripts received, and included in the Special Issue.

## 2. An Overview of Published Articles

The study by Karim et al. (Contribution 1) identified genetic causes of developmental delay (DD), congenital malformations (CMs), and intellectual disability (ID) in Saudi children using array comparative genomic hybridization (aCGH) to detect disease-associated copy number variation (CNV). They collected 63 children with DD/CM/ID and detected chromosomal abnormalities in 24 patients, including variant CNVs of different pathogenicity and/or uncertain significance in 19 patients, and aneuploidy in 5 patients. The diagnostic yield of aCGH (28%) is higher than that of conventional karyotyping (15.9%), but the two can be complementary [7]. The paper also revealed the presence of some rare pathogenic CNVs in Saudi’s pediatric patients suffering DD/CM/ID. This is the first comprehensive study based on array CGH in Saudi Arabia and the first to report the existence of the very rare pathogenic CNVs/genes in the region.

The article by Chou et al. (Contribution 2) investigated the long-term outcomes of three children with neonatal seizures suffering from PACS2 mutations, and critically reviewed this syndrome in the literature. PACS2 is a rare genetic disorder affecting brain development attributed to an early infantile development and epileptic encephalopathy (EIDEE) [8], with the characters of neonatal seizures, facial deformities, speech delays and abnormalities in the posterior fossa. Whole-exome sequencing (WES) showed de novo heterozygous missense variants in p.Glu209Lys of PACS2 gene in 32 patients except 1 patient. Therefore, genetic testing of PACS2 mutations is essential for the early diagnosis and personalized management of PACS2-associated EIDEE.

The article by Malta et al. (Contribution 3) provided a comprehensive review of holoprosencephaly (HPE), a rare genetic disorder that affects brain development and causes facial abnormalities. HPE is the commonest prosencephalic malformation in human beings with the characters of structural abnormalities of the brain due to cleavage failure of prosencephalon. There are typically three subtypes of HPE, that is, the alobar, semilobar, and lobar holoprosencephaly radiologically, which may be associated with varying degrees of craniofacial defects from cyclopia/proboscis, ethmocephaly, cebocephaly, premaxillary agenesis to hypotelorism/cleft lip and/or palate. HPE is caused by genetic and non-genetic factors. The genetic etiologies of HPE may include chromosomal and monogenic disorders. Patients with syndromic HPE are often associated with chromosomal abnormalities or monogenic disorders, however, nonsyndromic HPE is more likely to have a single gene cause [9]. About 25–50% of HPE patients had chromosomal abnormalities, while 10–14% showed CNVs, and 18–25% revealed monogenic disorders [10]. The regulation of sonic hedgehog (SHH) pathway is assumed to be involved in the pathophysiological basis of HPE.

A review by Chang et al. (Contribution 4) detailed the advances in epilepsy genomics and its applications for genetic testing in children with developmental and epileptic encephalopathy (DEE), a disorder in which cognitive function is affected by seizures and the neurobiological processes underlying epilepsy [11]. This article provided the overview of recent major advances in epilepsy genomics focusing on DEE in children. The advances of clinical testing using gene panel, exome, and genomics have uncovered the diagnosis of many genetic epilepsies, including DEE. Exome sequencing (ES) may cover most coding parts of causing genes and is proven a powerful tool to identify pathogenic variants, including incidentally discovered variants [12]. The article also provided a flowchart to outline the DEE investigation process.

Ko and Chen’s narrative review (Contribution 5) provided a whole genome sequencing model for children with unexplained GDD and ID. Overall in clinical practice, singleton or trio ES is more cost-effective for studying patients with GDD/ID [13]. Therefore, the authors proposed an evaluation algorithm for unexplained GDD/ID, with the suggestion of ES as the first-tier of evaluation. Furthermore, re-analysis of ES data can increase the diagnostic yield by 10 to 16 percent [14], and it is recommended to re-assess inconclusive results after six to 12 months.

The review by AlMutiri et al. (Contribution 6) discussed the different genetic tests currently available and explored the latest evidence and recommendations for genetic assessment in children with nonsyndromic GDD and ID. As a fact, GDD/ID can be divided to nonsyndromic GDD/ID, where GDD/ID is the predominant clinical picture, and syndromic GDD/ID, in which there are other clinical manifestations or comorbidities. Recently, genetic testing has become the most important diagnostic tool for clinicians to evaluate children with nonsyndromic GDD/ID. Previously, most society guidelines recommended chromosomal microarray (CMA) testing as the first-line test [15], with a diagnostic yield of between 10 and 20 percent for mild ID and 20 to 30 percent for moderate and severe ID. CMA can nearly replace the use of karyotyping. Besides, fragile X syndrome (FXS) is also recommended as the first-line testing for all patients with GDD/ID. One meta-analysis found that ES had a diagnostic yield of 36 percent in NDD [16], and periodic re-analysis of ES over time may also improve diagnostic yields. In 2021, the ACMG guidelines recommended ES as a first-/second-tier testing for children with GDD/ID [17]. The recent introduce of genome sequencing (GS) is an NGS method to analyze the sequence of the majority of DNA in the whole genome. The major advantage of GS is its ability to examine intron regions, to detect structural arrangements, and the mitochondria, compared to ES [18].

## 3. Conclusions

This special issue includes several articles devoted to genetic diagnosis in children with developmental delay, reflecting the rapid development or progress in genetic diagnosis in this field in recent decades. As summarized in Table 1, these contributions cover a large area of genetic diagnostic tools available in clinical practice. The main limitation of this issue is that the published papers are insufficient to cover all types of DD, with Contribution 1 identifying genetic causes of DD, CM, and ID in a developing area, while Contributions 2 and 3 detailing two specific encephalopathic entities. Others are review articles on the past and current status of genetic diagnostic testing in children with various status of DEE and GDD/ID. Nonetheless, this issue outlines the forward direction.

In summary, the choice of genetic testing depends primarily on a careful history taking, including family and developmental history, and detailed physical examination. If these findings are correlated to an acquired etiology, genetic testing may be not necessary. If a specific genetic cause or syndrome is suspected, appropriate targeted genetic testing for that syndrome is recommended. If GDD/ID refers to a nonsyndromic etiology, the choice of genetic testing depends primarily on the availability of genetic testing in the region or era. In general, chromosomal microarrays (CMAs) are the first-tier diagnostic genetic testing for patients with GDD/ID. The second-tier genetic testing may include next-generation sequencing of a comprehensive/polygenic GDD/ID panel or trio clinical exome or whole exome sequencing. Re-analysis in ES every 1–3 years can be attempted for negative ES. Further investigation using GS or segregation analysis may be considered. Other molecular techniques such as transcriptomics, metabolomics, proteomics are yet to be studied in the future.

## Figures and Tables

**Table 1:** Analysis of the published contributions in the Special Issue.

No.	Focus of Patients	Genetic Investigations	Type of Research
1.	DD, CM, ID	CMA	Case series survey
2.	Neonatal seizure	ES	Case reports/literature review
	(PAC2 mutation)		
3.	Holoprosencephaly	CK, CMA, TGP, ES	Systemic literature review
4.	DEE	CMA, TGP, ES	Systemic literature review
5.	GDD, ID	CMA, TGP, ES, GS	Narrative review
6.	Nonsyndromic GDD	CMA/FXS (1st tier)	Systemic literature review
		ES (2nd tier)	
		GS (further)	

CK: chromosome karyotyping; CM: congenital malformation; CMA: chromosomal microarray; DD: developmental delay; DEE: developmental and epileptic encephalopathy; ES: exome sequencing; FXS: Fragile X syndrome testing; GDD: global developmental delay; GS: genome sequencing; ID: intellectual disability; TGP: Targeted gene panel.

## References

[1] American Psychiatric Association (2013). Diagnostic and Statistical Manual of Mental Disorders DSM-5.

[2] Michelson D.J., Clark R.D. (2020). Optimizing genetic diagnosis of neurodevelopmental disorders in the clinical setting. Clin. Lab. Med..

[3] Bélanger S.A., Caron J. (2018). Evaluation of the child with global developmental delay and intellectual disability. Paediatr. Child Health.

[4] Gillentine M.A., Wang T., Eichler E.E. (2022). Estimating the prevalence of de novo monogenic neurodevelopmental disorders from large cohort studies. Biomedicines.

[5] Aspromonte M.C., Bellini M., Gasparini A., Carraro M., Bettella E., Polli R., Cesca F., Bigoni S., Boni S., Carlet O. (2019). Characterization of intellectual disability and autism comorbidity through gene panel sequencing. Hum. Mutat..

[6] Han J.Y., Jang J.H., Park J., Lee I.G. (2018). Targeted next-generation sequencing of Korean patients with developmental delay and/or intellectual disability. Front. Pediatr..

[7] Mithyantha R., Kneen R., McCann E., Gladstone M. (2017). Current evidence-based recommendations on investigating children with global developmental delay. Arch. Dis. Child..

[8] Zuberi S.M., Wirrell E., Yozawitz E., Wilmshurst J.M., Specchio N., Riney K., Pressler R., Auvin S., Samia P., Hirsch E. (2022). ILAE classification and definition of epilepsy syndromes with onset in neonates and infants: Position statement by the ILAE Task Force on Nosology and Definitions. Epilepsia.

[9] Ramakrishnan S., Gupta V. (2022). Holoprosencephaly. StatPearls.

[10] Dubourg C., Kim A., Watrin E., de Tayrac M., Odent S., David V., Dupé V. (2018). Recent advances in understanding inheritance of holoprosencephaly. Am. J. Med. Genet. C Semin. Med. Genet..

[11] Sharika Raga S., Specchio N., Rheims S., Wilmshurst J.M. (2021). Developmental and epileptic encephalopathies: Recognition and approaches to care. Epileptic Disord.

[12] Hebbar M., Mefford H.C. (2020). Recent advances in epilepsy genomics and genetic testing. F1000Research.

[13] Mercer T.R., Xu J., Mason C.E., Tong W., on behalf of the MAQC/SEQC2 Consortium (2021). The Sequencing Quality Control 2 study: Establishing community standards for sequencing in precision medicine. Genome Biol..

[14] Li J., Gao K., Yan H., Xiangwei W., Liu N., Wang T., Xu H., Lin Z., Xie H., Wang J. (2019). Reanalysis of whole exome sequencing data in patients with epilepsy and intellectual disability/mental retardation. Gene.

[15] Moeschler J.B., Shevell M. (2014). Committee on genetics comprehensive evaluation of the child with intellectual disability or global developmental delays. Pediatrics.

[16] Srivastava S., Love-Nichols J.A., Dies K.A., Ledbetter D.H., Martin C.L., Chung W.K., Firth H.V., Frazier T., Hansen R.L., Prock L. (2019). Meta-analysis and multidisciplinary consensus statement: Exome sequencing is a first-tier clinical diagnostic test for individuals with neurodevelopmental disorders. Genet. Med..

[17] Manickam K., McClain M.R., Demmer L.A., Biswas S., Kearney H.M., Malinowski J., Massingham L.J., Miller D., Yu T.W., Hisama F.M. (2021). Exome and genome sequencing for pediatric patients with congenital anomalies or intellectual disability: An evidence-based clinical guideline of the American College of Medical Genetics and Genomics (ACMG). Genet. Med..

[18] Sun Y., Peng J., Liang D., Ye X., Xu N., Chen L., Yan D., Zhang H., Xiao B., Qiu W. (2022). Genome sequencing demonstrates high diagnostic yield in children with undiagnosed global developmental delay/intellectual disability: A prospective study. Hum. Mutat..

